# Supplementary material for: End Sequence Analysis Toolkit (ESAT) expands the extractable information from single-cell RNA-seq data
Source: Genome Res. 2016 Oct;26(10):1397–410. doi: 10.1101/gr.207902.116 (PMC5052061; doi:10.1101/gr.207902.116)
Supplement: Supplemental Material [file supp_gr.207902.116_Supp_Materials.tar.gz › ESAT-esat_v0.1/doc/html/functions_p.html]

ESAT: Class Members


ESAT


- Main Page
- Related Pages
- Packages
- Classes
- Files

- Class List
- Class Index
- Class Hierarchy
- Class Members

- All
- Functions
- Variables
- Enumerator

- a
- b
- c
- d
- e
- f
- g
- h
- i
- j
- k
- l
- m
- n
- o
- p
- q
- r
- s
- t
- u
- v
- w
- z


All Classes Namespaces Files Functions Variables Enumerator Pages

Here is a list of all class members with links to the classes they belong to:

### - p -

- PaGEJava()
  : broad.pda.geneexpression.PaGEJava
- Pair()
  : broad.core.datastructures.Pair< T1 >
  , jaligner.formats.Pair
- PAIRED
  : broad.pda.seq.graph.ChromosomeWithBubblesJGraphT.EdgeSourceType
- PAIRED\_END
  : broad.pda.seq.rap.CollectAnnotationEnrichments
  , broad.pda.seq.rap.GenomeScoringProgram
  , broad.pda.seq.rap.rna.TranscriptomeScoringProgram
- PAIRED\_END\_EXTENSION
  : umms.core.writers.PairedEndWriter
- PAIRED\_SUPPORT
  : broad.pda.seq.graph.ChromosomeWithBubblesJGraphT.EdgeSourceType
- PairedEndReader()
  : umms.core.readers.PairedEndReader
- PairedEndReadIterator()
  : broad.pda.samtools.PairedEndReadIterator
- PairedEndWriter()
  : umms.core.writers.PairedEndWriter
- PairedReadAlignment()
  : umms.core.alignment.PairedReadAlignment
- pairedTStat()
  : broad.core.math.Statistics
- pairwiseNonoverlappingIgnoreStrand()
  : umms.core.utils.AnnotationUtils
- parse()
  : broad.core.annotation.AnnotationReader< T extends GenomicAnnotation >
  , broad.core.annotation.BasicAnnotationReader
  , broad.core.annotation.BasicGenomicAnnotationReader
  , broad.core.annotation.BEDGraphReader
  , broad.core.annotation.BEDReader
  , broad.core.annotation.GFFReader
  , broad.core.annotation.RepeatMaskerAnnotationReader
  , broad.core.annotation.ShortBEDReader
  , broad.core.parser.CommandLineParser
  , broad.core.parser.StringParser
  , broad.core.primer3.ComputeMIRScore
  , broad.core.sequence.FastaParser
  , broad.core.sequence.FastaQualityParser
  , broad.core.siphy.TreeScalerIO
  , broad.pda.datastructures.AlignmentsReader
  , broad.pda.geneexpression.agilent.AgilentToGCT
  , broad.pda.seq.fastq.FastqParser
  , jaligner.util.SequenceParser
- parseAgilentExperiment()
  : broad.pda.geneexpression.agilent.AgilentUtils
- parseAgilentExperimentGlobalStats()
  : broad.pda.geneexpression.agilent.AgilentUtils
- parseAndWrite()
  : broad.pda.tools.PSLXToSAM
- parseCDT()
  : broad.core.util.ParseGCTFile
- parseCHIPFile()
  : broad.core.util.GMTParser
- parseChipFile()
  : broad.core.util.ParseGCTFile
- parseCHIPFileByName()
  : broad.core.util.GMTParser
- parseCLS()
  : broad.core.util.ParseGCTFile
- parseCLSGroups()
  : broad.core.util.ParseGCTFile
- parseControlSamples()
  : broad.pda.geneexpression.agilent.AgilentUtils
- ParseException()
  : broad.core.error.ParseException
- parseExperimentInfoFile()
  : broad.pda.geneexpression.agilent.AgilentUtils
- parseExperimentInfoFileToGroup()
  : broad.pda.geneexpression.agilent.AgilentUtils
- parseExperimentInfoFileToGroups()
  : broad.pda.geneexpression.agilent.AgilentUtils
- parseExperimentInfoFileToName()
  : broad.pda.geneexpression.agilent.AgilentUtils
- parseExperimentInfoGroups()
  : broad.pda.geneexpression.agilent.AgilentUtils
- parseExperimentInfoNonControls()
  : broad.pda.geneexpression.agilent.AgilentUtils
- parseGeneList()
  : broad.core.util.ParseGCTFile
- ParseGeneSetFile()
  : broad.core.util.GMTParser
- ParseGMTFile()
  : broad.core.util.GMTParser
- parseGMXFile()
  : broad.core.util.GMTParser
- parseGoRawFiles()
  : broad.pda.gene.functionalAnnotation
- parseMaskFiles()
  : broad.pda.seq.segmentation.ContinuousDataAlignmentModel
- parseNumericCls()
  : broad.core.util.ParseGCTFile
- parseRawPointRegions()
  : broad.core.annotation.BasicGenomicAnnotationReader
- parseRawRegions()
  : broad.core.annotation.BasicGenomicAnnotationReader
- passedQC()
  : broad.pda.geneexpression.ExpressionExperimentInfo
- passes()
  : broad.pda.seq.segmentation.AlignmentDataModel
  , broad.pda.seq.segmentation.AlignmentDataModelStats
  , broad.pda.seq.segmentation.GenericAlignmentDataModel
  , broad.pda.seq.segmentation.ReadFilter< T >
- passesCutoff()
  : broad.pda.seq.segmentation.AlignmentDataModel
  , broad.pda.seq.segmentation.GenericAlignmentDataModel
- Path()
  : broad.pda.seq.graph.Path
- pathToGene()
  : umms.core.scripture.OrientedChromosomeTranscriptGraph
- PCT\_MASKED\_ALLOWED
  : broad.pda.seq.rap.GenomeCommandLineProgram
- pctOverlapWithRef()
  : broad.pda.annotation.Locus
- pearsonCorrelation()
  : broad.core.math.Statistics
- pearsonDistance()
  : broad.core.math.Statistics
- percentGC()
  : broad.core.primer3.PrimerUtils
  , broad.core.sequence.SequenceRegion
- percentGenomeOverlapping()
  : umms.core.annotation.Gene
- percentOverlapping()
  : umms.core.annotation.Gene
- PERMUTATION\_ATTEMPTS
  : umms.core.coordinatesystem.GenomicSpace
  , umms.core.coordinatesystem.TranscriptInGenomicSpace
  , umms.core.coordinatesystem.TranscriptomeSpace
- PermutationDistribution()
  : broad.core.math.PermutationDistribution
- PermutationNotFoundException()
  : umms.core.coordinatesystem.PermutationNotFoundException
- PERMUTATIONS
  : broad.pda.seq.rap.BuildRatioNullDistribution
  , broad.pda.seq.rap.CollectAnnotationEnrichments
  , broad.pda.seq.rap.ShuffleBAM
  , broad.pda.seq.rap.ShuffleBED
- permute()
  : broad.core.siphy.TreeScalerIO
- PERMUTE
  : broad.pda.seq.rap.CollectAnnotationEnrichments
- permuteAndAssignFDR()
  : broad.pda.differentialExpression.Permutations
- permuteAnnotation()
  : umms.core.coordinatesystem.CoordinateSpace
  , umms.core.coordinatesystem.GenomicSpace
  , umms.core.coordinatesystem.MaskedGenomicSpace
  , umms.core.coordinatesystem.TranscriptInGenomicSpace
  , umms.core.coordinatesystem.TranscriptomeSpace
- permuteAnnotationsOnChromosome()
  : broad.core.annotation.AnnotationReader< T extends GenomicAnnotation >
- permuteColumns()
  : broad.core.motif.PositionWeightMatrix
  , broad.core.multiplealignment.MultipleAlignment
- permuteData()
  : broad.pda.differentialExpression.Permutations
- permuteDataByGroupSize()
  : broad.pda.differentialExpression.Permutations
- permuteRegions()
  : broad.pda.seq.segmentation.ContinuousDataAlignmentModel
- PhastconsReader()
  : broad.core.siphy.PhastconsReader
- piEMIteration()
  : broad.core.siphy.EvolutionaryModel
- PiHMM()
  : broad.core.siphy.PiHMM
- Pileup()
  : broad.pda.seq.clip.Pileup
- placeInTree()
  : broad.core.datastructures.IntervalTree< V >
- plot()
  : broad.core.annotation.BasicGenomicAnnotationReader
- plus()
  : Jama.Matrix
- plusEquals()
  : Jama.Matrix
- POINT\_MATH
  : broad.pda.seq.rap.DistanceToBED
- POINT\_PATTERN
  : broad.core.annotation.BasicGenomicAnnotationReader
- poisson()
  : broad.core.math.ScanStatistics
- PoissonEnrichmentScore()
  : umms.core.model.score.PoissonEnrichmentScore
- PositionWeightColumn()
  : broad.core.motif.PositionWeightColumn
- PositionWeightMatrix()
  : broad.core.motif.PositionWeightMatrix
- PositionWeightMatrixIO()
  : broad.core.motif.PositionWeightMatrixIO
- PositionWeightMatrixModel()
  : broad.core.siphy.PositionWeightMatrixModel
- POSITIVE
  : umms.core.annotation.Annotation.Strand
- pow()
  : broad.core.datastructures.MatrixWithHeaders
- PrecomputeDataAlignmentStats()
  : broad.pda.seq.segmentation.PrecomputeDataAlignmentStats
- PreprocessDataSet()
  : broad.pda.geneexpression.PreprocessDataSet
- Primer3Exception()
  : broad.core.primer3.Primer3Exception
- print()
  : broad.core.motif.PositionWeightColumn
  , Jama.Matrix
- printHelpMessage()
  : broad.core.parser.CommandLineParser
- printKmer()
  : broad.core.motif.PositionWeightMatrix
- printProb()
  : broad.core.motif.PositionWeightColumn
- printRandModels()
  : broad.pda.gene.transcriptsNullModel
- printRandNeighborsInfo()
  : broad.pda.gene.NeighborsNullModel
- printRefinedWeightMatrix()
  : broad.core.motif.PositionWeightMatrix
- ProcessDataset()
  : broad.pda.geneexpression.agilent.analysis.ProcessDataset
- processOneAnnotationSet()
  : broad.pda.seq.rap.CollectAnnotationEnrichments
- processWindow()
  : umms.core.model.score.WindowProcessor< S extends WindowScore >
- PROTEIN
  : jaligner.Sequence
- pruneAndPeel()
  : broad.core.siphy.EvolutionaryModel
- pruneTree()
  : broad.core.siphy.ConservationUtils
  , broad.core.siphy.EvolutionaryModel
- PSLRecord()
  : umms.core.annotation.PSLRecord
- put()
  : broad.core.datastructures.IntervalTree< V >
- pvalue()
  : broad.core.math.Statistics
- pValueCutoffScan
  : broad.pda.seq.protection.TwoSampleScanSkellamPeakCaller
- pValueCutoffSkellam
  : broad.pda.seq.protection.TwoSampleScanSkellamPeakCaller


---

Generated on Fri Aug 15 2014 15:11:16 for ESAT by  

 1.8.7
